# Feasibility of Non-Anesthesiologist-Administered Propofol Sedation for Emergency Endoscopic Retrograde Cholangiopancreatography

**DOI:** 10.1155/2015/685476

**Published:** 2015-03-29

**Authors:** Nobuhito Ikeuchi, Takao Itoi, Takuji Gotoda, Chika Kusano, Shin Kono, Kentaro Kamada, Takayoshi Tsuchiya, Naoyuki Tominaga, Shuntaro Mukai, Fuminori Moriyasu

**Affiliations:** ^1^Department of Gastroenterology and Hepatology, Tokyo Medical University, 6-7-1 Nishishinjuku, Shinjuku-ku, Tokyo 160-0023, Japan; ^2^Department of Gastroenterology, Yuri Kumiai General Hospital, 38 Iego Kawaguchi-aza Yurihonjyo-shi, Akita 015-8511, Japan

## Abstract

*Background*. The safety of non-anesthesiologist-administered propofol (NAAP) sedation in emergent endoscopic retrograde cholangiopancreatography (ERCP) has not been fully clarified. Thus, the aim of this study was to assess the safety of NAAP sedation in emergent ERCP. *Materials and Methods*. We retrospectively analyzed 182 consecutive patients who had obstructive jaundice and who underwent ERCP under NAAP sedation. The patients were divided into Group A (with mild acute cholangitis or without acute cholangitis) and Group B (moderate or severe acute cholangitis). And technical safety and adverse events were assessed. *Results*. The adverse events were hypoxia (31 cases), hypotension (26 cases), and bradycardia (2 cases). There was no significant difference in the rate of each adverse event of hypoxia and bradycardia in either group. Although the rate of transient hypotension associated in Group B was higher than that in Group A, it was immediately improved with conservative treatment. Moreover, there were no patients who showed delayed awakening, or who developed other complications. *Conclusions*. In conclusion, NAAP sedation is feasible even in emergent ERCP. Although some transient adverse events (e.g., hypotension) were observed, no serious adverse events occurred. Thus, propofol can be used in emergent ERCP but careful monitoring is mandatory.

## 1. Introduction

Conscious sedation is an essential element of advanced endoscopic procedures in selected institutes where general anesthesia is not commonly used. Aside from conventional endoscopic gastroduodenoscopy, endoscopic retrograde cholangiopancreatography- (ERCP-) related procedures may cause discomfort and stress in patients undergoing unpleasant and prolonged procedures [1]. One of the key factors determining the success of the procedure in patients is the administration of either moderate or deep sedation [2].

Propofol is an anesthetic that has been routinely used for anesthesia since 1984 [3]. Many researchers have reported the safety and efficacy of propofol administration in endoscopic procedures. This is especially relevant as the use of sedation has increased in gastrointestinal endoscopy over the last decade [4]. Moreover, the safety and efficacy of propofol sedation not only in observational endoscopic procedures but also in more complicated endoscopic procedures such as endoscopic submucosal dissection have been reported [5–7]. The increasing use of propofol lies in its excellent amnestic effect, rapid onset of action, and short duration of action. Propofol is metabolized mainly in the liver and most patients who require ERCP-related procedures have liver dysfunction due to obstructive jaundice. Although there are some reports regarding the safety of propofol administration during ERCP procedures [8–16], there is apparently no study regarding the safety of propofol administration in patients with a severe general condition receiving limited emergent ERCP for moderate or severe acute cholangitis. The safety of non-anesthesiologist-administered propofol (NAAP) sedation in endoscopic procedures, but not during emergent ERCP, has been reported. Thus, in the present study, we retrospectively assessed the feasibility of NAAP sedation in emergent ERCP.

## 2. Materials and Methods

### 2.1. Patients

We enrolled 182 consecutive patients who had obstructive jaundice and who underwent ERCP under propofol (1% Diprivan Injection-Kit; AstraZeneca, Cheshire, UK) sedation between July 2011 and October 2013 at Yuri Kumiai General Hospital, Japan.

Acute cholangitis is classified into 3 groups according to severity on the basis of the updated 2013 Tokyo guidelines [17, 18] for the management of acute cholangitis and acute cholecystitis. Emergent drainage is recommended in moderate and severe acute cholangitis. The severity assessment criteria for acute cholangitis are as follows [19].

*TG13 Severity Assessment Criteria for Acute Cholangitis*

*Grade III (Severe) Acute Cholangitis.* “Grade III” acute cholangitis is defined as acute cholangitis that is associated with the onset of dysfunction in at least one of any of the following organs/systems:

1. cardiovascular dysfunction hypotension requiring dopamine ≧5 *μ*g/kg per min, or any dose of norepinephrine,
2. neurological dysfunction disturbance of consciousness,
3. respiratory dysfunction PaO_2_/FiO_2_ ratio <300,
4. renal dysfunction oliguria, serum creatinine >2.0 mg/dL,
5. hepatic dysfunction PT-INR >1.5,
6. hematological dysfunction platelet count <100,000/mm^3^.

*Grade II (Moderate) Acute Cholangitis.* “Grade II” acute cholangitis is associated with any two of the following conditions:

1. abnormal WBC count (12,000/mm^3^, <4,000/mm^3^),
2. high fever (≧39°C),
3. age (≧75 years old),
4. hyperbilirubinemia (total bilirubin ≧ 5 mg/dL),
5. hypoalbuminemia (<STD × 0.7) (STD: lower limit of normal value; reproduced from [19] with permission of Springer Science).

*Grade I (Mild) Acute Cholangitis.* “Grade I” acute cholangitis does not meet the criteria of “Grade III (severe)” or “Grade II (moderate)” acute cholangitis at initial diagnosis.

*Notes.* Early diagnosis, early biliary drainage and/or treatment for etiology, and antimicrobial administration are fundamental treatments for acute cholangitis classified not only as Grade III (severe) and Grade II (moderate) but also Grade I (mild).

Therefore, it is recommended that patients with acute cholangitis who do not respond to the initial medical treatment (general supportive care and antimicrobial therapy) undergo early biliary drainage or treatment for etiology.

The patients were classified into Group A or Group B. Group A consisted of patients with mild acute cholangitis or without acute cholangitis who underwent elective ERCP (control group). Group B consisted of patients with a more severe general condition and who required emergent ERCP, namely, patients with severe or moderate acute cholangitis. The procedure time, details of propofol administration, the patient's resedated condition when returning to the ward, blood pressure, oxygen saturation, and heart rate during ERCP were retrospectively reviewed for all the patients. Adverse events, procedure time, and details of propofol administration were investigated. Patients were excluded from the study if they were <18 years old or had a history of sulfite, egg, soybean, or propofol allergies or did not provide informed consent.

### 2.2. Medication

Local pharyngeal anesthesia was performed using an 8% topical lidocaine spray before the intravenous administration of the sedative. Patients received a slow initial intravenous bolus of propofol given at 0.5 mg/kg/10 seconds. Additional intravenous boluses of propofol given at 0.5 mg/kg were slowly administered until sedation, as determined by a Ramsay sedation score [20] of 5 to 6. After each bolus infusion, a waiting period of typically 30 to 60 seconds was observed to assess whether the drug had completely taken effect before a decision was made to administer the next bolus. An automatic infusion pump was used to perform a continuous infusion of 2–5 mg/kg/hr to maintain the same level of sedation. The specific objective was to maintain the sedation level of a patient between moderate (the patient responds properly to verbal commands either given alone or accompanied by light tactile stimulation) and deep (the patient cannot be easily aroused but may respond properly to repeated or painful stimulation). All patients received 15 mg of pentazocine as an analgesic agent at the start of the ERCP and at 60-minute intervals thereafter during the procedure.

When a patient showed signs of discomfort or exhibited restlessness following verbal stimulation, an additional 10 mg of propofol was given as a bolus injection and the maintenance infusion rate was increased by 1 mg/kg/hr as resedation. Conversely, if an adverse event occurred, such as hypotension with a systolic blood pressure (SBP) of <80 mmHg or an oxygen desaturation of <90%, the maintenance dose was reduced by 1 mg/kg/hr. Propofol infusion was continued until endoscope removal. The delayed awakening of propofol is defined as the state of sedation for 15 minutes after the cessation of propofol administration [21]. All medications were administered by gastroenterologists who did not participate directly in the ERCP. Japanese clinical internship programs have mandated a specialty anesthetic training including propofol sedation. Thus, all gastroenterologists in Japan have received special training for sedation with propofol.

### 2.3. Monitoring

Patients received supplemental oxygen (2 L/min) by nasal cannula in the endoscopy room as their vital signs and oxygen saturation were continuously monitored and recorded every 5 minutes using a standard three-lead electrocardiogram, pulse oximetry, and automatic blood pressure equipment. Chest excursion and respiratory rates were monitored visually, and consciousness levels were assessed initially after the induction of sedation using the Ramsay sedation score. Patients were discharged from the endoscopy room following the ERCP after confirming that they were fully awake and responding to questions and that their vital signs were stable.

### 2.4. Management of Adverse Events

Adverse events were considered to be indicated by a decline in oxygen saturation to <90% or an SBP of <80 mmHg. If a patient developed oxygen desaturation of <90% for more than 10 seconds, supplemental oxygen was used to immediately increase the oxygen flow until the saturation level was >95%. If supplemental oxygen failed to improve the patient's oxygenation condition within 3 minutes, ERCP and sedation were interrupted to secure the airway.

When hypotension was recognized as SBP < 80 mmHg every 5 minutes of standard observation, blood pressure was immediately rechecked. When SBP < 80 mmHg was confirmed, the rate of intravenous drip was immediately increased from 100 to 150 mL/hr, and decreased the propofol infusion rate was decreased by 1 mg/kg/hr. If supplemental SBP did not improve the patient's SBP condition within 3 minites, ephedrine administered at 8 mg by bolus intravenous injection.

## 3. Statistical Analyses

Normally distributed data were expressed as mean ± SD. Statistical significance was analyzed using the chi-square test or Fisher's exact probability test and Aspin-Welch's *t*-test. Statistically significant differences were denoted by *P* < 0.05. Statistical analysis was performed using SPSS (ATMS Co. Ltd., Tokyo, Japan).

## 4. Results

### 4.1. Baseline Characteristics

A total of 182 patients (men/women = 90/92) receiving ERCP with propofol sedation were registered. The patients were classified into 2 groups: Group A (*n* = 149) and Group B (*n* = 33). A large number of patients who required ERCP were elderly patients (mean age: 75.4 ± 10.3 years). The mean age of the patients in Group B, who had a more severe general condition, was more advanced than that of the patients in Group A. In fact, 63 (34.6%) patients had past medical histories involving the heart, blood vessels, lung, or kidney. The American Society of Anesthesiologists Physical Status (ASA-PS) classification [22] III/IV rate was 58.2% (Table 1).

Details of the laboratory data of each group are shown in Table 2. The white blood cell (WBC) count and albumin level in Group A were significantly higher than those in Group B. The mean values of C-reactive protein (CRP) and the indicators used in the liver function test, namely, aspartate aminotransferase (AST), alanine aminotransferase (ALT), *γ*-glutamyl transpeptidase (*γ*-GTP), and total bilirubin (T-Bil), were significantly higher than the reference ranges. However, there was no significant difference in the levels of CRP, AST, ALT, *γ*-GTP, and T-Bil between Group A and Group B.

### 4.2. Details of Propofol Administration

The mean procedure time was 49.1 ± 0.02 minutes. The mean amount of propofol used was 157.0 ± 93.2 mg. There was no patient in whom the procedure was suspended for insufficient sedation. The first bolus induction dose, number of times of additional bolus injection, additional bolus injection dose, average maintenance dose (mg/kg/hr), and total infusion dose during the ERCP procedure are shown in Table 3. There was no statistically significant difference in the abovementioned items without the first bolus induction dose between Group A and Group B.

### 4.3. Adverse Events in ERCP

There were no patients who showed delayed awakening after discharge from the operating room, or who developed other complications. All adverse events associated with cardiopulmonary functions were temporary. There were no severe adverse events associated with cardiopulmonary functions with sequelae. Moreover, there was no patient who required a vasopressor or endotracheal intubation. The adverse events associated with cardiopulmonary functions included hypoxia in 31 patients, hypotension in 26 patients, and bradycardia in 2 patients. There was no significant difference in the rate of each adverse event of hypoxia and bradycardia associated with cardiopulmonary functions in each group. On the other hand, the rate of adverse event of hypotension associated with cardiopulmonary functions in emergent ERCP in Group B was higher than that in Group A (Table 4).

## 5. Discussion

Acute cholangitis due to obstructive jaundice will require emergency ERCP. As the mortality rate of severe acute cholangitis is high at 64.7% [23], some patients with such severe condition will require ERCP.

Propofol has a high rate of conjugation with serum albumin, shows a high perfusion-limited clearance, and undergoes fast metabolism in the liver. Thus, patients who have liver dysfunction have decreased propofol metabolism. This results in the maintenance of high blood levels which inhibit cardiopulmonary functions likely to be manifested as hypoxia, hypotension, or bradycardia. Although there were several limitations in this investigation being retrospective in nature and a single-center study, our preliminary assessment indicated the safety of NAAP sedation in emergent ERCP.

The total rates of adverse events associated with cardiopulmonary functions manifested as hypoxia, hypotension, and bradycardia were 20.4%, 17.1%, and 1.3%, respectively. Notably, the rates of these adverse events in Group B in which the patients had a more severe general condition were 9.1%, 27.3%, and 0%, respectively.

Over the last 2 decades, the safety of propofol administration in ERCP has been wellreported (Table 5) [8–16]. However, the rates of adverse events associated with cardiopulmonary functions were 5.1%–22.3%, 2.6%–20.0%, and 0%–5.1% for hypoxia, hypotension, and bradycardia, respectively, and there was no severe adverse event (Table 4). Although the rate of hypotension in Group B, in which the patients had a more severe general condition, appeared higher than the rates in previous reports, all adverse event cases of hypotension appeared after bolus infusion. However, those adverse events were immediately improved without the appearance of a serious adverse event of hypotension by increasing the rate of the intravenous drip and decreasing the propofol infusion rate by 1 mg/kg/hr. When emergent ERCP is performed after propofol bolus infusion, caution should be taken to prevent a decrease in blood pressure.

Propofol is metabolized mainly in the liver and has a high rate of the conjugation with serum albumin. Propofol that is not conjugated with serum albumin exerts an anesthetic effect. Therefore, liver dysfunction and hypoalbuminemia may reduce propofol clearance in the liver [24, 25]. Our study showed that the levels of the serum indicators of liver function were higher than the reference levels, although the difference between the 2 groups was not statistically significant. The serum albumin level in Group B was lower than that in Group A. There was no serious cardiopulmonary adverse event in either group.

A previous meta-analysis indicated that propofol sedation in ERCP was not associated with any increased risk of complications [26]. Servin et al. [27] compared the pharmacokinetics of propofol infusions in patients with liver cirrhosis and in those without hepatic dysfunction and found that the total body clearance was not reduced significantly. Although the volume of distribution of propofol at the steady state was significantly greater in patients with liver cirrhosis than in control patients, there was no significant difference in the terminal elimination half-life. Thus, Servin et al. [27] concluded that the pharmacokinetics of propofol given by infusion to maintain general anaesthesia was not affected markedly by moderate liver cirrhosis. These results in conjunction with ours suggest that propofol administration to patients with hypoalbuminemia or liver dysfunction in emergent ERCP may be tolerable if it is performed with sufficient surveillance and care.

In many countries, it is recommended that propofol should be used by anesthesiologists or that it must be administered under the direct supervision of anesthesiologists. However, propofol is not always available, particularly in small hospitals. Recently, the concept of NAAP sedation has emerged [28–30]. However, the US Food and Drug Administration states that only anesthesia-trained personnel can administer propofol for sedation procedures because of the significant risks associated with propofol. In several countries, propofol sedation in an endoscopic procedure must be administered only by an anesthesiologist. Rex et al. reported that NAAP is estimated to save $5.3 million for each life per year [29], and it increases the healthcare cost coverage. Thus, NAAP sedation may substantially contribute to savings for healthcare cost coverage. However, the safety of NAAP sedation in emergent ERCP has rarely been investigated. In fact, there is apparently no report on NAAP sedation during emergent ERCP. In the present study, all of the patients underwent NAAP sedation. NAAP sedation safety was assessed even in emergent ERCP. Overall, the total percentage of cardiopulmonary adverse events was permissible in reference to previous reports. Most published studies showed that propofol was administered in ERCP using the nonanesthesiologist procedure. Reports in the literature regarding the safety of NAAP sedation in ERCP involving 20 patients with ASA-PS classification III and higher showed only 2 cardiopulmonary adverse events, namely, a minor adverse event requiring bag-mask ventilation and a major adverse event necessitating mechanical ventilation via endotracheal intubation [16]. Both adverse events were in accordance with ASA-PS classification III and were managed via the nonanesthesiologist approach. There were no patients who required cardiopulmonary resuscitation and admission to the intensive care unit. Sedation-related deaths were not observed in the study.

In conclusion, NAAP sedation is feasible even in emergent ERCP. Although some transient adverse events associated with cardiopulmonary functions (e.g., hypotension) were observed, no serious adverse events occurred. Thus, propofol may be used in emergent ERCP but careful monitoring is mandatory.

## Figures and Tables

**Table 1:** Baseline characteristics of 182 patients receiving ERCP with propofol sedation.

	Total	Group A	Group B	*P* value
Number of cases	182	149	33	
Sex (male/female)	90/92	77/72	13/20	
Age (mean ± SD)	75.4 ± 10.3	74.1 ± 10.3	82.2 ± 7.1	0.0019
Body mass index	22.7 ± 3.7	23.0 ± 3.8	21.2 ± 3.0	0.0060
Body weight (kg)	54.3 ± 13.1	55.3 ± 13.3	49.9 ± 11.0	0.0223
ASA-PS classification				
I/II (%)	76 (41.8)	65 (43.6)	11 (33.3)	0.2781
III/IV (%)	106 (58.2)	84 (56.4)	22 (66.6)	0.2781
Underlying diseases				
OSA (%)	2 (7.4)	2 (7.4)	0	0.7999
Cardiovascular disease (%)	29 (15.9)	23 (15.4)	6 (18.2)	0.6966
Respiratory disease (%)	9 (5.0)	6 (4.0)	3 (9.1)	0.4411
Renal disease (%)	7 (3.9)	6 (4.0)	1 (3.0)	0.8174
Vascular disease (%)	18 (10.0)	16 (10.7)	2 (6.1)	0.4154

ERCP: endoscopic retrograde cholangiopancreatography.

Group A: control group consisting of patients with mild acute cholangitis or without acute cholangitis.

Group B: group requiring emergent ERCP with severe or moderate acute cholangitis.

ASA-PS classification: American Society of Anesthesiologists Physical Status classification.

SD: standard deviation.

OSA: obstructive sleep apnea.

**Table 2:** Laboratory examination data of Group A and Group B.

	Reference range	Total average (*n* = 182)	Group A (*n* = 149)	Group B (*n* = 33)	*P* value
WBC (/*µ*L)	2700–8800	7819.3 ± 4816.8	6238.6 ± 2213.6	15353.3 ± 6734.2	<0.001
Platelet (/*µ*L)	14–34	21.4 ± 8.9	22.1 ± 8.5	18.6 ± 10.7	0.1009
AST (U/L)	8–38	136.0 ± 171.3	124.4 ± 164.3	197.4 ± 198.7	0.0884
ALT (U/L)	4–44	146.9 ± 184.9	146.9 ± 182.0	150.7 ± 206.2	0.9490
*γ*-GTP (U/L)	16–73	480.0 ± 609.6	484.6 ± 634.6	462.0 ± 487.9	0.7724
T-Bil (mg/dL)	0.2–1.2	4.0 ± 5.7	3.8 ± 5.9	4.6 ± 5.1	0.4991
ALB (g/dL)	3.9–5.3	3.1 ± 0.7	3.2 ± 0.7	2.7 ± 0.8	0.0006
CRP (mg/dL)	<0.3	11.2 ± 73.8	10.6 ± 81.0	15.1 ± 7.1	0.6181

Group A: control group consisting of patients with mild acute cholangitis or without acute cholangitis.

Group B: more severe general condition group consisting of patients with severe or moderate acute cholangitis.

WBC: white blood cells, AST: aspartate aminotransferase, ALT: alanine aminotransferase, *γ*-GTP: *γ*-glutamyl transpeptidase, T-Bil: total bilirubin, ALB: albumin, and CRP: C-reactive protein.

**Table 3:** Details of propofol administration.

	Total average	Group A (*n* = 149)	Group B (*n* = 33)	*P* value
Procedure time (minutes)	49.1 ± 0.02	49.0 ± 0.02	49.9 ± 0.02	0.2102
First bolus induction dose (mg)	29.2 ± 11.7	29.6 ± 11.7	25.9 ± 9.0	0.0391
Number of times of additional bolus injection	1.5 ± 1.8	1.5 ± 1.6	1.7 ± 2.3	0.5707
Additional bolus injection dose (mg)	34.0 ± 39.4	34.0 ± 38.4	33.8 ± 7.3	0.9862
Average maintenance dose (mg/kg/hr)	0.014 ± 0.008	0.014 ± 0.009	0.015 ± 0.007	0.9610
Total infusion dose (mg)	157.0 ± 93.2	158.9 ± 89.2	145.6 ± 112.5	0.5240

Group A: control group consisting of patients with mild acute cholangitis or without acute cholangitis.

Group B: more severe general condition group consisting of patients with severe or moderate acute cholangitis.

**Table 4:** Adverse events associated with cardiopulmonary functions in each group.

	Total	Group A (*n* = 149)	Group B (*n* = 33)	*P* value
Hypoxia (%)	31/182 (17.0)	28/149 (18.8)	3/33 (9.1)	0.1798
Hypotension (%)	26/182 (14.3)	17/149 (11.4)	9/33 (27.3%)	0.0185
Bradycardia (%)	2/182 (1.1)	2 /149 (1.3)	0/149 (0)	0.7999

Total (%)	59/182 (32.4)	47/149 (31.5)	12/33 (36.4)	0.7416

Group A: control group consisting of patients with mild acute cholangitis or without acute cholangitis.

Group B: more severe general condition group consisting of patients with severe or moderate acute cholangitis.

**Table 5:** Summary of reports in the literature on ERCP with propofol sedation.

Author	Year	Sedation	Administrator	Number of cases	Mean age	Adverse event (%)	Hypoxia <90% (%)	Hypotension SBP <90 mmHg (%)	Bradycardia HR <50/min (%)
Wehrmann et al. [8]	1999	Propofol	Physician	99	63.6 ± 23.3	NA	11 (11.1)	7 (7.1)	5 (5.1)
Krugliak et al. [9]	2000	Propofol	Anesthesiologist	15	56.8 ± 12.5	0	0	0	NA
Jung et al. [10]	2000	Propofol	Anesthesiologist	39	62	NA	2 (5.1)	1 (2.6)	NA
Vargo et al. [11]	2002	Propofol	Physician	38	52.9 ± 2.4	20 (52.6)	14 (36.8)	6 (15.8)	0
Chen et al. [12]	2005	Propofol	NA	35	53.89 ± 17.12	15 (42.9)	2 (5.7)	7 (20.0)	0
Riphaus et al. [13]	2005	Propofol	Physician	75	83.7 ± 7.8	NA	8 (10.7)	6 (8.0)	3 (4.0)
Kongkam et al. [14]	2008	Propofol	ACLS trained physician, gastroenterologist	67	52.31	NA	15 (22.3)	6 (9.0)	2 (3.0)
Angsuwatcharakon et al. [15]	2012	Balanced-propofol	Endoscopic nurse	103	59.56 ± 13.65	NA	NA	14 (13.6)	1 (1.0)
Khan et al. [16]	2014	Propofol	BLS and ACLS trained physician, gastroenterologist	156	54.39 ± 17.0	2 (1.3)	2 (1.3)	0	0
Present study	2014	Propofol	Endoscopist	182	75.4 ± 10.3	59 (38.8)	31 (20.4)	26 (17.1)	2 (1.3)

ERCP: endoscopic retrograde cholangiopancreatography.

ACLS: advanced cardiac life support.

BLS: basic life support.

NA: not available.
